# Molecular diagnosis of cytogenetic abnormalities in patients with schizophrenia

**DOI:** 10.1192/j.eurpsy.2022.734

**Published:** 2022-09-01

**Authors:** M. Bar Shai, A. Peleg, T. Zalozhin

**Affiliations:** 1 Carmel Medical Center, Clalit Healthcare Service, Psychiatry, Genetics, Haifa, Israel; 2 Genetics, Carmel Medical Center, Clalit Healthcare Service, Haifa, Israel, Genetics, Carmel Medical Center, Clalit Healthcare Service, Haifa, Israel, Haifa, Israel

**Keywords:** Schizophrenia, Cromosomal abnormalities, CMA, diagnosis

## Abstract

**Introduction:**

Schizophrenia is a severe and chronic disorder causing significant disability and functional decline. Schizophrenia is a polygenic disease, with about 100 monogenic sites and 11 sites of chromosomal deletions / duplications involved in its pathogenesis identified. It is a pleiotropic disease, with causative genetic changes leading to multiple symptoms, including bipolar disorder, autism spectrum disorders, ADHD, mental retardation and epilepsy. The chromosomal microarray (CMA) technology detects submicroscopic chromosomal changes, which are involved in neurodevelopmental disorders, and are subject to prenatal diagnosis. Pathological findings in CMA are detected in 10-20% of patients with neurodevelopmental disorders and can contribute significantly to medical follow-up, prognosis assessment, influence treatment choice, and allow prenatal diagnosis. Preliminary studies in schizophrenia identified pathological CMA findings in 10–30% of patients.

**Objectives:**

CMA testing of schizophrenia patients to detect genetic changes causing the disease.

**Methods:**

Recruitment of schizophrenia patients from the Haifa and Western Galilee districts of Clalit, genetic counseling in Carmel Hospital, CMA testing of the consenting patients.

**Results:**

Schizophrenia patients with and without neurodevelopmental disorders underwent CMA analysis, with the findings of significant chromosomal submicroscopic disorders (such as 22q11 microdeletion, among others) in 30% of the patients, providing the explanation for the patients’ symptoms and enabling specific medical follow-up and adjusted pharmacological treatment.

**Conclusions:**

CMA can be used in diagnosing schizophrenia, assessing prognosis, adjusting pharmacological treatment and follow-up and providing genetic counseling including prenatal diagnosis, as in cases neurodevelopmental disorders. The findings support the application of CMA as part of a routine procedures in schizophrenia.

**Disclosure:**

No significant relationships.

